# A Day’s Practice

**Published:** 1884-01

**Authors:** N. S. Jenkins

**Affiliations:** Dresden, Germany


### Independent Practitioner.

Vol. V. January, 1884. No. 1.

### Original Communications.

### A DAY'S PRACTICE.

BY N. S. JENKINS, D. D. S., DRESDEN, GERMANY.

READ BEFORE THE AMERICAN DENTAL SOCIETY OF EUROPE, AT COLOGNE.

On a pleasant Monday morning in the month of May, an American dentist in a Continental town entered his office with a cheerful heart. The day before his pastor had preached most eloquently upon the trials of Job, concluding his discourse by saying: "Rejoice then, my brethren, not only over this great example of the glorious triumph of the all-patient Job, but also that your weak faith can never, under the modern dispensation, be called to meet such trials."

With these words still ringing in his ears, this good doctor, as I was saying, entered his office with a cheerful heart; but his young lady assistant met him with a troubled air, holding a number of letters in her hand. "Every moment is occupied to-day, Doctor, but several people have sent word that they must see you immediately."

Madame Wheinoffska writes: "Something troubles me upstairs in the background of my mouth. I have dreadful pains, and cannot rest a moment. I will come at your house at twelve, for even if it is your breakfast, I am sure you will be so amiable to see me."

97/135

"Well if she is suffering pain I must give her my lunch time. What else?"

"Madame de Katkoff says: 'When passing through Paris three weeks ago, I consulted your compatriot, Dr. Bull, and he did make me a large *plombe.* Yesterday it quite fell out while eating a little bird. Please be so kind to take me this day, for I must depart for Russia immediately.'" "Well, let her come at a quarter before eleven; that's during Smith Thompkins' hour, but he is a good fellow, and will gladly give up part of his time to a lady in distress. Anything else?" "All the others can wait except this one. 'Lady Buzz will call on Mr. Blank at about twelve, to-day, to have a tooth stuffed.'" "Well I can only let her come for an examination. Now then, show in the half-past eight o'clock patient.

Second Lieutenant Von Zarsky enters with a nervous alacrity of movement, very unlike the stately ceremonious manner that he cultivates so successfully in society. His spurs jingle, his sabre clanks, he drops his helmet while offering to shake hands with the doctor, and when the fraeulein picks it up for him he murmurs something about not having slept all night for anxiety as to what this hour was to bring forth.

"You see, much honored Doctor," says this brave son of Mars, "I can stand anything, only pain of the teeth not. This is a peculiar susceptibility of mine, and I beg you to be considerate of my weakness." A moment's examination shows a broad horseshoe-shaped denture, the second and third molars curving inwards after the manner of the South-German jaw, suggestive of that far-off time when Slavic and Teutonic blood was mingled in the *Voelkerwanderung.* There were numerous cavities in the molars, and the first superior bicuspids were broken down and abscessed. These roots the doctor decided to treat and pivot, but seeing what unreasonable terror had taken possession of his patient, he found that he must first gain his confidence. "There are many operations to be made for you. They will not be painful, but rather tedious. To-day I will make but one, that you may see how easily it goes, and then hereafter you will come without anxiety." Then with very gentle touch, and carefully studied deliberation of movement, with the delicate use of wheel, drill, and excavator, allowing the patient to see each instrument and to understand its use, a crown cavity is slowly excavated and quickly filled with three cylinders of soft gold, packed with lateral pressure and condensed by the mallet. The patient is conquered. The excited flush on his cheek gives place to his natural color. The great drops of perspiration are wiped from his brow, and do not return. His spasmodic breathing gives place to his slow natural rythm, and he no longer evinces an uncontrollable desire to spend forty seconds in every minute in rinsing his mouth. As the Lieutenant departs with words of real gratitude on his lips, and readily assumes again his formal dignity of manner, the doctor knows that hereafter this patient will give him but little trouble.

The nine o'clock patient now enters. She is a delicate American girl, who is suspected of having spinal disease. A glance at her mouth shows rather small, bluish white teeth, with very thin enamel, somewhat stained by the mineral waters she has been drinking, and a great number of solidly packed and beautifully finished gold fillings, some of which, where the frail edges seem to have been malleted too severely, threaten to necessitate repair at no distant day. But she has come to-day to have the right superior second bicuspid refilled. "Do you use hard or soft gold, doctor?" she enquires. "Sometimes one, sometimes both, sometimes neither. But why do you ask?" "My dentist uses only hard gold, and the electric mallet, and he told me to be sure and not let any one in Europe touch my teeth who did not work exactly as he did." "Then you must wait until you go back to him, for there are not two good dentists in the world who use exactly the same methods." "But I can't do that, for I am not going home for two years, and I think the neuralgia from which I have been suffering may come from this tooth."

Examination shows a beautiful contour filling in the posterior approximal surface and crown of the bicuspid, loose, with some frail edges of enamel broken away, and the whole tooth blackened by decay under the filling.

A touch of the chisel removes the weak point of crown enamel which retained the filling in place, and as it falls into the hands of the operator the full extent of the disaster is revealed. Caries had attacked, not only the bicuspid, but also a saucer-shaped depression high upon the anterior approximal surface of the molar, at the bottom of which cavity palpitated an exposed pulp. The doctor's first impulse was to treat the exposed pulp only, at this sitting, but the patient begged to have the bicuspid filled if possible, saying: "I sat three hours to have this tooth filled, two years ago, and as I feel so strong this morning I should like to have it done now, since I cannot reckon on my health. I do not at all mind pain. I am well used to suffering." The doctor inwardly commented upon the barbarity which could give such a patient a three hours' sitting, but said nothing and commenced his work. With files, and corundum disks and points, he quickly cut down the edges, approximal and crown, to a perfectly smooth surface, sacrificing perhaps a sixth part of the breadth of the bicuspid, but gaining walls strong enough to endure the force of mastication. The root, into which the discoloration had not extended, was well filled with oxy-chloride of zinc. The rubber dam was applied to both teeth. The pulp of the molar was touched with creosote and capped with Weston's non-irritating cement, while the bicuspid was most carefully excavated until every particle of discolored dentine was removed, and then filled with phosphate of zinc. As soon as this filling had hardened sufficiently, good retaining points were made in root and crown, and various portions of the phosphate were removed until a good anchorage was everywhere secured, except at the buccal edge, where only a thin shaving of the phosphate was taken away, and then amalgam was carefully packed over both the phosphate and the Weston cement. The color of the bicuspid was restored. The frail walls were supported by the cementing power of the phosphate, and the cement in both teeth was protected from disintegration by the amalgam, which was left to be polished at a later sitting. It had required a little over an hour of rapid but most careful work to make these perfectly practical operations, at the least possible strain upon the patient, but as she went away she was heard to say to her maid: "I have no confidence in operations not made with gold, and which do not hurt more than that."

Smith Thompkins came at ten. He had been a sickly boy, and his teeth had been neglected until pain sent him to a dentist, who chloroformed him and extracted all the upper molars on the right side, and all the lower molars on the left. He was now forty years old, and was manifesting gouty proclivities. Mucous and saliva gave an acid reaction, and wherever his bicuspids and incisors touched, and at any weak spot that he could not reach with the tooth brush there was abrasion of the enamel and wasting of the dentine, until it was necessary to build out extensively with gold. To-day the point of a cuspid was to be restored, and it was desirable to work rapidly, as the doctor had determined to send him off at a quarter before eleven, to receive Madame de Katkoff. But Smith Thompkins was a man of views, and to-day he insisted for the tenth time upon giving the dentist his theory of decay, which he believed in his case to be the result of taking rhubarb in his third year, and he wanted to examine his own teeth and give a minute description of how each one had been treated, and to tell how chloroform affected him, and what his father's maternal aunt had died of, and how his great-grandfather at the age of ninety-eight had never had toothache in his life, his longevity being probably due to New England rum, and where was the best place to buy cigars, and who would be the next President of the United States, until the doctor in the very midst of a syllable slipped on the rubber dam, and told his loquacious patient that he must be very quiet or the consequences might be serious. For a few seconds ether spray was thrown on the abnormally sensitive tooth, and then with a chisel-sharpened burr-drill, retaining points radiating away from the pulp were bored beneath the glassy surface, and the points connected by a narrow channel, grooved by the dental engine. Sponge gold was used in the most shallow of the retaining points, then annealed Williams' pellets, and the point was rapidly assuming its proper proportions, when a card was brought in. "Please, dear Mr. Doctor, see me and my little boy for an instant at once, on a matter of greatest importance." The doctor glanced at the hour. Ten minutes remained before the appointment with Madame de Katkoff, and he needed all this time for Smith Thompkins.

"Well, perhaps it *is* only for an instant; show her into the 'Co.'" (We called the Consultation Office *Co.* for short.)

Assuring himself that the rubber dam was firmly attached, and cautioning his office girl to watch it, he told Smith Thompkins that he would allow him to rest a moment, and darted off to the Co. There he found a Frenchwoman in a state of great excitement, driving a pretty boy of seven nearly distracted by her frantic appeals to be tranquil. "Be tranquil, dearest Adolph, for the love of God be tranquil. The good doctor will do nothing to you; he will only look at the naughty tooth and give you some nice medicine for it." "Well, what is the matter?" "Oh dear, Mr. Doctor," (speaking in a language which the child did not understand,) "one of his lower front teeth is loose, and it ought to be taken out, as it moves so much that he can't eat, and I am so afraid, for he is such a delicate child. Please take it out quick, but tell him you won't touch it." "Have I not told you before that it is both wrong and foolish to deceive a child? Were I to do so now how could I manage him when there was something really serious to be done? Come here, my boy, and let me see what the matter is—Bah! that is nothing. The tooth is so loose that I can teach you how to take it out yourself. Give me your finger, then press so on one side while I press on the other, and there it is, all right. Good bye," and the doctor runs back to his office, leaving the fond mother in an ecstacy of delight over the tranquility of her Adolph. He arrives not a moment too soon. Smith Thompkins has been telling the office girl about his estate on the banks of the Mississippi, where he has spent over one hundred thousand dollars on embellishments, which he now regrets, and what a superior and very extensive education his wife has enjoyed, and what is the price of tenderloin beefsteak at St. Louis, and how the duke of Noland once said to him; "My dear Mr. Smith Thompkins, all you Americans have the grand air." Just then the doctor put his finger on the rubber dam and barely saved an inundation. A few minutes of rapid work and the contour is completed, and Smith Thompkins consents to come again next week for the polishing, but pauses with his hand on the door to tell the doctor how they cure hams in Norway, and to ask the address of the best hairdresser, and to call his banker a grasping Jew, and to say that the American Consul who can't play poker is unfit for his post, when he is gently precipitated into the reception room where five ladies all rise simultaneously and say in three different languages, "Good morning, Doctor!"

Smith Thompkins appalled into silence glides out, while the doctor, selecting by instinct the Russian, begs Madame de Katkoff to enter his office, and sends out his fraeulein, armed with the appointment book, to negotiate treaties of peace with the other ladies. Madame de Katkoff relates with great volubility her experience with "Dr." Bull, of Paris, and ends by taking out of her purse a flattened lead shot, saying triumphantly, "There, I thought the fillings of American dentists never fell out." The doctor examined the little missile and replied, "The gentleman you call Dr. Bull is neither an American nor a dentist. He is an English mechanic, and the diploma you saw hanging in his office was bought in Philadelphia. He is a person to whom no dentist ever sends a patient, unless it be for a simple piece of mechanical work, which he does very well. Still this is not his filling which I hold in my hand, but the occasion of death to that little bird about which you wrote to me." Mr. Bull's filling was then examined and found to be an already somewhat disintegrated phosphate, but promising to hold tolerably for six months to come, when Madame de Katkoff was to be again in Paris, and she was dismissed with a Paris address, where there is no bogus diploma hanging on the wall, but where the tooth will be filled so as to last her a lifetime.

As she departs the fraeulein announces; "the Duchess, due at eleven arrived before her time, and has been waiting impatiently; there is also a Sister bringing a card from Prof. Gutman"; (the head of the city hospital). The doctor reads: "My dear Colleague; one of our nurses has been suffering from obstinate neuralgia and I begin to suspect that it arises from the teeth. Please give her necessary treatment and oblige," etc.

The doctor enters the reception room and bowing to the duchess says: "Your Highness, punctuality is the politeness of princes—and of doctors; but here is a lady whom a colleague has just sent to me in great suffering; of course you will permit me to see her first." "Um; yes, certainly; only I hope it will not be for long." "Not a moment longer than will be absolutely necessary to relieve her."

But it is an obscure case. All the molars on the left side have been extracted. The pain which comes periodically and is increased by extremes of temperature, is wholly on the right side. The teeth seem to be without blemish, and are not sensitive to percussion. The doctor is almost inclined to think that here is at last a case of inflammation from pulpstone. But after a time the electric light discloses a slight opacity between the superior first and second molars. A touch of a point of ice at this place increases the pain instantly; chisel and revolving disk are at once brought into play. The brave little woman although worn by three weeks of suffering does not wince, while the doctor, as tenderly and quickly as possible, makes a practicable approach to two very obscure cavities and finds exposed pulps, so inflamed that the only treatment is extirpation. "Perhaps it is better to extract the tooth," says the sister, "for I have only this week. Next Monday I am ordered to another station." He has not a free hour during the week, but he brushes away the temptation like a cobweb. "No, Sister, I cannot let you go to your self-denying labors with no molars to masticate your much-needed food, and I should like to punch the head of the man who took out those on the other side of the mouth. I shall take you every day between other patients, until I get you in order. Good bye; you will sleep to-night."

The Duchess enters. Her maid of honor removes her bonnet, and takes charge of her gloves, and opens her fan, and humbly subsides into a corner while the duchess speaks.

"It is six months since I have been in town, but four weeks ago I noticed a discoloration on these teeth," (showing her left inferior bicuspids). "I went to the learned Prof. Knowall while passing through Vienna, and he assured me that it was a mushroom growth, for which the only cure is treatment with an acid which would dissolve the lime in the tooth, and enable the bacteria to bore holes into the nerve. But still it looks ugly, and I thought perhaps you could give me a good advice how to get rid of these mushrooms without so great injury to the teeth." "But, your Highness, that is only tartar. It is a calcareous and not a mushroom growth, and will disappear at once upon being touched with a scaler and polished with pumice. Look and see how it disappears." "Yes, but Dr. Knowall showed me under the microscope how these mushrooms were formed, and how the bacteria bored into the nerve, and assured me that it was of no use to remove them except with an acid which would destroy both mushroom and tooth, but which would not affect the bacteria; and I can't look so ugly, and I can't loose my teeth, and even if you have made them white now I know those awful mushrooms will grow again, and then what shall I do?" "Your Highness, I have already had the honor to inform you that this stain which I have removed is tartar, and not a mushroom growth. It has come upon these teeth only because they are protected from the tooth-brush by the prominence of the cuspid and first molar. It is only necessary to use a small brush, and to be sure to reach these depressed surfaces with it, to keep them in perfect order." "But if this mushroom growth attacks my other teeth and the bacteria bore into my nerves everywhere, what shall I do then?" "Consult Prof. Knowall again. He will have a more consoling theory by that time." But this duchess was only to be comforted by a new prescription for a tooth-powder, warranted to be sure death to mushrooms and bacteria, and as she marched away the doctor felt a momentary comfort in the thought that his fraeulein had written her down in the appointment book as the *Dutschess.*

Noon had arrived, and with it Madame Wheinoffska. Tall, graceful, vivacious, the very picture of health, she at once assumed the most distressed air upon entering the office, and said mournfully: "Dearest Doctor, I am suffering frightfully. I can neither eat, nor sleep, nor talk, nor sing. I fear I am going mad with this dreadful pain. At one moment it is in my cheek, then in my teeth, then in my brain. But don't hurt me. For the love of Heaven have compassion on my distracted nerves. It may be rheumatism of the head ; may it not?" "How long have you suffered?" "Three days." "Does either heat or cold affect you?" "No." "Nor the wind?" "No, but I cannot bite. It is agony when I try to eat." A short investigation discloses a fishbone sticking in the gum between an upper bicuspid and molar. After much groaning and many threats of an intention to faint, the bone is drawn out and an immediate cure effected. "There is no danger of a fistula from the operation, I hope," says the bright creature as she trips away.

Lady Buzz is now received. "I will only detain you a moment, Mr. Blank. Just poke a little stuffing into this tooth if you please, and be so good as to look at the teeth of my daughter and my niece." "I am very sorry, but there is no time to-day to make an operation, but I will see what is to be done and make a future appointment." "But I should be very sorry to have endured this tropical heat for nothing." (it was sixty-five degrees Fahrenheit in the shade). "My dentist in London lets me come when I like, and every three months he puts in a little stuffing, and it never takes him five minutes." "After a few more such treatments there will be no more teeth to stuff. I cannot fill that tooth properly in less than an hour, but if I fill it for you, it will be a permanent operation." "Well then, please look at these two young ladies, and tell me why my niece does not have such good teeth as my daughter." "Because she had a Scotchman for a grandfather." "How do you know that?" "Because she has a long and narrow jaw, thin enamel and bluish white teeth, long and thin like the jaw, and dentine so soft that a small cavity easily penetrates to the pulp. Your daughter, on the other hand, has a broad square jaw, and firmly set yellowish white teeth, Anglo-Saxon to the very pulp; hard as flint, and capable of standing a treatment when once decayed, of being 'stuffed' in five minutes. All the unbolted flour and oatmeal in the world will not build up the teeth of your niece so that they will not need frequent care at the hands of the dentist ; but your daughter may have her teeth treated by a barber's apprentice, and they will still live to tell the tale."

There are yet ten minutes left before the next patient is due, and the doctor takes a bite of lunch, while dictating a letter and two telegrams, and rushes back to his office to receive Madame de Patoffska and her daughter. They are strangers. *Polen ous der Polakai.* Both are trembling violently. There are tears in the mother's eyes and voice as she says: "We have just arrived from Warsaw, according to your kind appointment, but I am afraid to no purpose. My daughter is so delicate and faints so easily that I fear she can never bear the pain of having her tooth treated. For Heaven's sake be careful, and do not give her pain, for she has never had anything done to her teeth before." After many preliminaries and frequent asseverations of her daughter's weakness and nervousness, (she appeared but for her fright to be in perfect health,) the patient is seated in the chair. The doctor took up a mouth mirror, but as he approached her mouth with it the patient caught at his hand and screamed. The mother, who was holding her left hand with averted head, groaned and cried: "Gently, dear Doctor, for God's sake, be merciful. Please, please, be careful of my poor weak child!" "But this is nonsense," said the doctor, sternly; "If you will be quiet I will examine your teeth and tell you what there is to do, and I will not hurt you. But if you cannot control yourself I have no choice but to send you away." "But we can't go away after coming so far, and we want the teeth put in order; there, see; she will be patient. Grasp my hand, you poor dear, and compose yourself."

At last, after many groans and writhings, and countless sighs, it is possible to discover that the patient has only a few small cavities in accessible situations, and a great abundance of tartar, irritating the gums and making the mouth most unsightly. The doctor says: "Now be reasonable; what I have to do for you will give you no pain, but you must be quiet unless you wish to hurt yourself. *I* won't hurt you!"

At that moment he is called to the Co. to change a wedge. Upon his return he finds mother and daughter on their knees, and praying before a great penwiper hanging high on his writing desk, bearing on its front a gorgeously worked representation of the

Father of his country. They rise, and the daughter being either strengthened by her devotions or comforted to find that it really doesn't hurt, gradually ceases to groan and cry, and only sobs convulsively from time to time, and kicks occasionally like a jumping-jack. After making a simple gold filling, the doctor polishes one front tooth to perfect whiteness, knowing that the patient will be more tractable if she can entertain a hope that all her teeth will eventually look like that.

As they depart, the mother enquires: "What Saint is that?" "The American Saint George, Madame! When his country was in danger of running down hill, he put the drag-on. He was cannonized in his time, but not by his Highness, the Pope."

Miss Daisy Miller is now announced. She comes in dragging Randolph by the arm. "Mother says she wants you to give Randolph gas, and pull out two lower teeth which hurt him when he eats candy. He had a lot taken out just before leaving New York, and candy only just now begins to hurt him again."

A glance shows that all the lower temporary molars have been taken out before their time; only the sixth year molars are left, and the lower jaw, having only a slight occlusion at the incisors, has begun to protrude. The sixth year molars are considerably decayed, but the pulps not exposed. "It will never do for this child to lose any more teeth. He can hardly eat as it is. These teeth must be filled, and then they will hurt him no more." "Mother don't believe in filling teeth which have ever hurt. A dentist put something in one of her teeth once to kill the nerve, and pounded in a filling the next day with a hammer, and after a week her face swelled up awful; and she just went and had all her upper teeth out, and hasn't had toothache since."

"Nevertheless I decline to take out Randolph's teeth. The child's troubles can be cured much more easily and certainly by filling than by extracting. No enlightened dentist now thinks of extracting teeth because they give pain." "Dear me; *my* dentist out West always takes out aching teeth, and at Colton's, in New York, they don't do anything else." "It is useless to continue this discussion. Shall I treat Master Randolph as I think best or not at all?" "Yes," cries Randolph, "I'd a heap rather have 'em filled than pulled out. You just shut up, Miss Daisy, or I'll tell the Doctor where you buy your back hair."

The rubber dam is quickly adjusted, and the work commenced, but the doctor finds Randolph very restless and inclined to weep at the least suggestion of pain, but at last, by a copious use of carbolic acid and working with very sharp excavators with the lightest and most delicate touch, one cavity is excavated, floored with agate cement, and filled with cohesive gold, and Randolph sent away to try if candy hurts that tooth any more.

Next comes Fraeulein Jagerson. Her father died in raging madness. She is tall, thin, angular, intensely nervous. Her teeth are attacked readily by white decay, and the most superficial cavity is sensitive to changes of temperature, to sweets, acids, or salt, and to the slightest touch. The doctor is tired, and he sighs as he remembers former sittings with this patient. To-day an approximal cavity on a superior first bicuspid is to be filled. Space had already been laboriously made during the previous week by the slow process of wedging with dry cotton, the only pressure that this patient can endure. With slow and gentle movement the doctor adjusts the rubber dam. The patient shrinks as the rubber touches her cheek, she groans as it is slipped over the teeth, she clasps her head with both hands as the ligatures are tied, she weeps as the cavity is dried and the air finds unobstructed access to the sensitive decay. With careful touch the doctor begins to smooth the rough margins with a thin file, while the tall form of the patient shakes and shudders like a galvanized frog. His patience is nearly exhausted, and he is on the point of a sharp remonstrance, when the memory of One who has borne our griefs and carried our sorrows comes to him, and with fresh compassion he tries anew to quiet and soothe his struggling patient. As on previous occasions, so here he finds that he can only excavate after a prolonged use of obtundents, and even then but by the exercise of the greatest care and most delicate manipulation. But at last the cavity is excavated, and filled with soft gold about the margins and cohesive gold in the center, and Fraeulein Jagerson is dismissed. The doctor rejoices to know that the next patient is a man. He has a left inferior wisdom tooth half erupted and already decayed, with a third of the cavity under the gum. He has such a sensitive throat and palate that he can bear no touch back of the second bicuspid, and so the cavity has to be dried and filled with great rapidity, as the use of any sort of napkin is here impossible. Four cylinders of tin and gold are used in this case, and although the filling suffers an inundation as the last cylinder is being packed, it is well condensed and promises to do good service.

Now come two treatment cases to be advanced a stage. Then a partial plate is placed in a mouth where syphilis and mercurial treatment have loosened the teeth and softened the gums so as to make its adjustment a matter of great difficulty, and the doctor is inclined to rebel that this man's sin should have laid upon his overtaxed nerves, with so much otherwise unnecessary trouble.

Last of all comes a girl of fourteen to have the right superior first molar extracted. This is a regulating case. The molar is decayed down to the gum, and slightly abscessed. It is desirable to remove it to obtain room in the crowded jaw. On a previous occasion a cavity half an inch deep had been bored in the palatine root, and a stout piece of rubber placed between the first and second molars to loosen the tooth. A screw is now inserted into a hole in the root, and with a simple movement the tooth is slipped out entire, leaving the thin alveola perfectly uninjured and ready to receive and firmly hold the crowded bicuspids.

The day's work is done. Every nerve palpitates with the strain of bearing other people's burdens so many hours, but the doctor hears the brisk step of his boy returning from school to go out on the last train to their summer house in the country with his papa, and his spirits revive,—when the card of Miss Rodgers is handed to him, with a request for a moment's interview. Miss Rodgers is a poor governess whom he treats out of charity. A superior lateral incisor, drilled and mutilated years ago in England, and which he has been holding together for the past three years with phosphate, has been broken off. The poor girl is in despair. The next morning she is to journey to Vienna to apply for a new situation, and cannot appear in such a plight. The doctor gazes wistfully at the western hills. "There are his young barbarians all at play." He remembers how his little girl said to him a few days ago; "Papa it would be so nice in the country if only we saw more of you." And then he thinks, twenty years from now my girl may need some one's compassion, and he sends word for the boy to go alone, and to say that he will drive out to a belated dinner, and begins to prepare a Bonwill crown for Miss Rodgers. But the gum is much absorbed, and the root rather short if ground down to the margin of the gum, and he must make a joint where no stain of amalgam can possibly shine through. So at last he decides to set the pivot in the crown that evening with amalgam, to leave it to harden during the night, and to come in half an hour earlier in the morning and set the pivot then in the root with phosphate. So the pivot is set, and the amalgam packed, and Miss Rodgers is dismissed for the night.

But as the doctor stretches his weary length in the carriage which is rapidly conveying him to his belated dinner, he thinks with new interest of the patriarch Job, and rejoices once more that under the modern dispensation neither patience nor faith are subjected to a strain greater than we can bear.

